# A new age for biomedical applications of Ribosome Inactivating Proteins (RIPs): from bioconjugate to nanoconstructs

**DOI:** 10.1186/s12929-016-0272-1

**Published:** 2016-07-20

**Authors:** Elio Pizzo, Antimo Di Maro

**Affiliations:** Department of Biology, University of Naples “Federico II”, Via Cintia, I-80126 Napoli, Italy; Department of Environmental, Biological and Pharmaceutical Sciences and Technologies (DiSTABiF), Second University of Naples, Via Vivaldi 43, 81100 Caserta, Italy

**Keywords:** Anticancer, Bioconiujates, Immunotoxins, Nanomaterials, Plant toxin, Ribosome-inactivating proteins

## Abstract

Ribosome-inactivating proteins (RIPs) are enzymes (3.2.2.22) that possess N-glycosilase activity that irreversibly inhibits protein synthesis. RIPs have been found in plants, fungi, algae, and bacteria; their biological role is still under investigation, even if it has been recognized their role in plant defence against predators and viruses. Nevertheless, several studies on these toxins have been performed to evaluate their applicability in the biomedical field making RIPs selectively toxic towards target cells. Indeed, these molecules are extensively used to produce chimeric biomolecules, such as immunotoxins or protein/peptides conjugates. However, to date, clinical use of most of these bioconiujates has been limited by toxicity and immunogenicity. More recently, material sciences have provided a wide range of nanomaterials to be used as excellent vehicles for toxin-delivery, since they are characterized by improved stability, solubility, and in vivo pharmacokinetics. This review discusses progresses in the development of RIPs bioconjugates, with particular attention to the recent use of nanomaterials, whose appropriate design opens up a broad range of different possibilities to the use of RIPs in novel therapeutic approaches in human diseases.

## Background

**Fig. 1:**
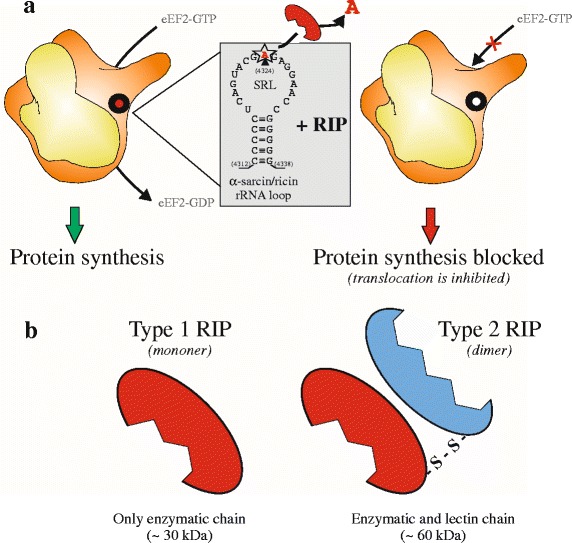
Enzymatic and structural properties of RIPs. **a** RIPs recognize α-sarcin/ricin loop (SRL), highlighted in grey, of rRNA 28S on ribosomes. Depurination of A_4234_ (see text) by N-glycosilase activity inhibits role of eukaryotic elongation factor 2 (eEF2) in the promotion of the GTP-dependent translocation of the nascent protein chain from the A-site to the P-site of the ribosome, with consequent arrest of protein synthesis. **b** Structural representation of type 1 RIPs (single enzymatic chain) and type 2 RIPs (dimeric protein consisting of an enzymatic chain and a lectin domain)

Ribosome-inactivating proteins (RIPs) belong to a class of enzymes identified in plants, fungi, algae, and bacteria. RIPs exhibit rRNA N-β-glycosilase activity, which leads to the cleavage of an adenine residue at a conserved site of the 28S rRNA [1], Fig. 1a. Cleavage of this single N-glycosidic bond is irreversible and interferes with the association between the elongation factors and ribosome, causing the inhibition of protein synthesis [2].

In addition to their cytotoxic effects, many RIPs have additional biological actions, not necessarily related to N-glycosilase activity, on cells and/or on organisms. Beyond that, it has been reported that some RIPs possess additional enzymatic activities on different substrates that include adenine polynucleotide glycosylase activity, phosphatase activity on lipids as well as chitinase, DNase and superoxide dismutase activities [3–5].

RIPs can be categorized into two groups, distinguishable in monomeric type 1 RIPs and dimeric type 2 RIPs. These latter are composed by a chain that exhibits the toxic rRNA N-glycosilase activity (A-chain) and by a lectin chain (B chain) as schematized in Fig. 1b. Accentuated toxicity of type 2 RIPs respect to type 1 RIPs is due to recognition, mediated by A-chain, of mammalian cell surface galactose moieties. The absence of the lectin chain significantly limits the access of type 1 RIPs into cells, determining a consequent lower cytotoxicity. Beyond these, some non-canonical RIPs, such as tetrameric ebulin [6, 7] or proteolytic activated maize b-32 [8] were also found.

Different reports have highlighted the existence of a close correlation between RIPs cytotoxicity and intracellular routing, which may vary between different cell types depending on: (i) expression of different types of binding molecules (ligands) on cell surface [9, 10]; (ii) sorting of RIP-ligand complexes to different compartments [11, 12]; (iii) availability of various pathways for the transport of the toxin to the cytosolic compartment [13–15].

These considerations are also corroborated by the existence of non-toxic RIPs, identified in some plants, that despite having N-glycosilase activity are non-toxic, since they are degraded as a result of an intracellular routing different from that of toxic type 2 RIPs [7, 16].

Generally, RIPs enter the cell by first binding to cell surface receptors, then crossing the cell membrane via endocytosis and, finally, translocate into the cytosol by an intracellular compartment. Type 2 RIPs (e.g. ricin), across the membrane via endocytosis, after binding to galactose moieties, and are delivered from Golgi network to the endoplasmic reticulum (ER) by retrograde vesicular transport. Once in the ER lumen, the A and B chains are dissociated and finally the active A chain portion is directed to the cytoplasm [11, 13].

On the contrary type 1 RIPs, devoid of lectin B-chain, once internalized, are delivered to the cytoplasm through a Golgi independent route, thereby making their uptake by cells more difficult [9, 14].

However, the possibility of selectively directing RIPs to target cells opens huge applications in medicine [17–19], as demonstrated by targeted RIP based toxic preparations used in a number of clinical trials and in a very large number of preclinical studies [20, 21].

## Review

### RIP based immunotoxins

**Fig. 2:**
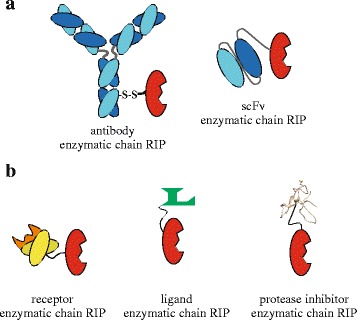
Putative RIP based conjugates. **a** ImmunoRIPs obtained fusing RIPs to antibody (*left*) or minibody, as scFv (*right*). **b** Alternative bioconiujates obtained fusing RIPs to receptors (*left*), cellular ligands (center) and protease inhibitors (*right*)

Chimeric molecules obtained combining antibody portion and specific toxins are known as immunotoxins: bifunctional macro-molecules that are based on intracellular toxin action to kill target cells [22]. The progress of recombinant antibody engineering and protein fusion technology has led to rapid expansion of drug-targeting devices with superior antigen binding and pharmacokinetic properties (Fig. 2a) [23]. Nowadays immunotoxins are considered powerful immune-bullets against cancer cells, in immune regulation and in the treatment of viral [24] or parasitic diseases [25].

Since methods of conjugations of antibodies to RIPs can theorically affect the activity of immunoconjugates in vivo, different critical strategies have been performed to maximize the release of active toxins in the target cells [26, 27].

One of most approaches used in RIP based immunoconjugates synthesis for tumor cells, the latter typically characterized by a hypoxic state induced by an enhanced activity of reductive enzymes, is based on the choice of reducible disulfide cross-linker between RIP moiety and antibodies. In this way redox state of target cell should able to induce a reduction of RIP based immunoconjugate after its cell internalization, with consequent release of type 2 catalytic active A chain or type 1 RIP.

An alternative approach, conceived for RIPs lacking free sulphydryls (e.g. gelonin, PAP, saporin etc.) and used also to carrier whole type 2 RIPs, was based on the use of specific cross-linker that bind reactive functional groups present on proteins as primary amines, carbonyls, carbohydrates and carboxylic acids generating new sulphydrylic groups. To carry out this strategy, conjugate preparation required a preliminary separate derivatization of both toxin and antibody; subsequently newly inserted groups on two molecules can react to produce, by disulfide bridges, a stable conjugate population [28]. Some of the most used heterobifunctional crosslinking agents for immunotoxin conjugation by S-S disulfide, are SPDP [N-succinimidyl 3-(2-pyridyldithio) propionate]; SATA, [*S*-(*N*-succinimidyl) thioacetate]; SMPT, [(*N*-succinimidyloxy carbonyl)-1-methyl-1-(2-pyridyldithio) toluene] [26, 28].

Nevertheless, any problems were related to these approaches, due to possible premature reduction of immunoconjugates, which induced negative effects as:

1. competition between free antibodies and immunoconjugates in the recognition of target sites; 2. decrease of intracellular cytotoxic RIPs; 3. increase of side effects on several organs (e.g. liver). Other forms of heterobifunctional crosslinkers to create toxic conjugates not restricted by the disulphide bridge requirement are based on the ability to create stable tioether linkages (S-C). SMCC, [N-succinimidyl 4-(maleimidomethy1) cyclohexanecarboxylate] and MBS [3-maleimidobenzoic acid N-hydroxysuccinimide ester] are frequently used to form not cleavable thioether bonds between type 2 RIPs and antibodies.

The first RIP based immunotoxins were generated by coupling a deglycosylated type 1 or an A-chain from type 2 RIP with a native antibody by using specific cross-linking agents reacting with cysteinyl residues of both molecules [29, 30]. Subsequently, novel RIP based immunotoxins were produced and characterized to improve pharmacokinetics and reduce side effects; they were obtained by continuous development of recombinant DNA techniques and optimization of expression systems, using yeast, bacteria, or eukaryotic cell cultures (CHO cells, insect cells, etc.). The design of these novel RIP based immunotoxins required two critical steps: (i) planning and construction of recombinant antibody fragments (with reduced molecular weight); improvement of expression and purification methodology [31, 32]. The production of these compact immunotoxins also fulfilled the aim to stabilize the toxin during its intracellular routing (delivery through endosomes-Golgi-lysosomes) preventing its proteolytic degradation [21].

On the basis of above described considerations, an excellent RIP prototype, used to build immunotoxins targeted to different malignant cells or solid tumors, was type 1 RIP saporin-S6. Indeed saporin-S6 immunoconjugates evidenced good structural and functional characteristics, such as high resistance to denaturation and proteolysis, as well as strong catalytic efficacy coupled to a very low cytotoxicity on normal cells [3, 9].

Advances in antibody technology to avoid heterogeneity, improve tumor penetration and reduce production complexity of immunotoxins, have enabled the development of new generations of RIP based immunoconjugates. Starting from monoclonal antibody to the smallest unit of immunoglobin molecules (e.g. single chain fragment variable - scFv) several RIP based immunotoxins have been designed against several targets and in most cases used in pre-clinical studies, leading to promising outcomes [20, 33–35].

It is noteworthy that a further technology was successfully applied to improve immunotoxin delivery to cells: photochemical internalization technology (PCI) [36]. This technology is based on amphiphilic photosensitizers, which accumulate in the endocytic membranes. Light exposure causes generation of ROS and subsequent increased permeability of the endocytic membranes, thus allowing improved trafficking of agents to the cytosol.

### Protein or peptides conjugated with RIPs

Members of RIPs have been either fused or chemically conjugated to different suitable carriers, such as cell-binding ligands, protease inhibitors, hormones, etc., in order to create specific bifunctional cytotoxic agents (Fig. 2b).

Even for design and production of these new types of conjugates, saporin-S6 was used as a prototype. Indeed, this type 1 RIP was successfully fused to urokinase receptor (uPAR) to obtain a very effective bifunctional chimeric molecule with a strong cytotoxicity, specifically to uPAR expressing cells, whereas the conjugate was found not to be effective on cell lines devoid of uPARs [37].

In alternative reports, as a carrier in the design of conjugates it was used transferrin, a protein involved in iron uptake by cells. Transferrin is an iron-binding glicoprotein able to carry two iron ions in the ferric form (Fe^3+^) into the cell, upon binding to its receptor. Transferrin receptor, widely distributed in different cell types, is usually overexpressed in malignant cells. Artificial conjugates consisting of RIPs (e.g. saporin-S6, ricin, etc.) conjugated to transferrin revealed, although by different mechanisms of intracellular routing, a selective cytotoxicity on various cancerous or malignant cell lines [9].

Transferrin receptor was used as malignant cell target also to build RIP based conjugates containing a peptide carrier. A meaningful example is represented by curcin, a broad cytotoxic type 1 RIP from the seeds of *Jatropa curcas* L.. As this protein is able to inhibit tumor cell proliferation and promote cell apoptosis, it was used to build a conjugate in which, to enhance the targeting of its anti-tumor ability, a transferrin receptor peptide (TfRBP), was fused with it. This peptide was screened by phage display technology [21] and found to be a strong affinity for tumor cells over-expressing the transferrin receptor. Resulting conjugate curcin-TfRBP9 was found to significantly inhibit the proliferation of HepG2 cells over-expressing transferrin receptors and to have lower inhibitory effects on SKBR-3 cells expressing transferrin receptors at low levels [21].

Other representative chimeric conjugates were obtained by using the gonadotropin-releasing hormone (GnRH) as a carrier, since potent GnRH agonists and antagonists were widely used to treat different kind of reproductive apparatus cancer. For this purpose, type 1 RIP Pokeweed Antiviral Protein (PAP) was used, since it has no toxicity to human sperm and on epithelial cells in the female genital tract [38]. Treatment of GnRH receptor-positive cells, as human endometrial, breast or prostate cells, with the conjugate GnRH-PAP resulted in dose dependent cytotoxicity, thus demonstrating that other conjugates hormone/RIP could be used to specifically deliver these toxins to cells that express appropriate hormone receptors [39].

Other interesting fusion constructs generated with RIPs involved gelonin, a type 1 RIP from *Gelonium multiflorum* L.. Gelonin based conjugates, obtained by fusing it with different carriers, such as a cytokine (e.g. BLyS – B lymphocyte stimulator), a transmembrane glycoprotein kinase (e.g. Her2-protein encoded by a proto-oncogene) or an angiogenic factor (e.g. VEGF- vascular endothelial growth factor), showed a selective toxic action on tumor cells and on solid tumors [40].

It has been widely reported that RIPs cytotoxicity depends not only on the intracellular routing, but also on the intrinsic resistance to proteolysis. Pioneering works carried out on ricin free A chain [41] and saporin-S6 [3] confirmed this hypothesis, because their mutants (obtained by replacing surface residues with lysine residues), despite not compromising their activity, structure, or stability, significantly enhanced their susceptibility to proteolytic degradation. Moreover, as it is not unusual to find inhibitory protease modules in a multi-domain protein, new approaches have been carried out to build RIPs based chimeric proteins containing type 1 RIPs and protease inhibitor domains to enhance resistance to proteolysis during their intracellular routing [42]. In this regard, recently it has been described the characterization of a bifunctional chimeric molecule composed by PD-L4 (a type 1 RIP isolated from *Phytolacca dioica* L. summer leaves [43]) and WSCI (a serine protease inhibitor isolated from endosperm of hexaploid seeds of *Triticum aestivum* L.) [44]. This recombinant construct showed intact intrinsic activity of both domains (e.g. enzymatic activity and inhibitory properties), and at the same time an enhanced selective cytotoxicity on murine tumor cells. Similar results have also been obtained by changing the anti-protease inhibitory properties of WSCI domain [45].

Finally, a special mention has to be done to the use of peptides as carrier of RIPs for the construction of conjugates. A novel conjugate was obtained by fusing MAP30, a type 1 RIP from *Momordica charantia* L. and HBD, a cell penetrating peptide identified in the heparin-binding domain of human superoxide dismutase [46]. This fusion construct revealed an enhanced selective cytotoxicity on different tumor cell lines thanks to an efficient uptake mediated by the peptide, the latter being a prototype of a new class of short basic peptides that are revolutionizing the way to deliver biomacromolecules.

A different peptide, a human 36-aa neuropeptide widely distributed in brain and peripheral tissues, named NPY, has been used to build a conjugate with saporin-S6. Authors reported that this conjugate selectively killed NPY receptor-expressing neurons and, for this reason, it has been used as a tool to study the central NPY neurocircuitry involved in feeding behaviors [47].

### RIPs conjugated with nanoparticles

In recent years, considerable efforts have been made in the field of biomaterials providing increasing numbers of platforms for the development of a wide range of smart materials to control the delivery and release of specific drugs [48–50]. For this purpose, synthetic nanomaterials, including liposomes, polymers and inorganic nanoparticles, have been designed [51–53] to provide improved stability, solubility, and in vivo pharmacokinetics.

Owing to their intriguing features, such as size, shape and biocompatibility, nanoparticles have been receiving increased attention in the area of nano-drug delivery systems, since a variety of drugs, proteins, antibodies, peptides, etc. can be conjugated with them [54].

**Fig. 3:**
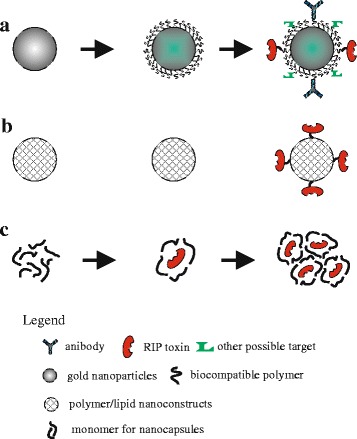
Innovative strategies to obtain a next generation of RIP based conjugates. **a** Metal nanoparticles consisting of RIPs and agents that drive resulting conjugate to cellular target; **b** Nanoconjugates obtained by combinatorial design of lipid like materials termed “*lipidoids*”; **c** Schematic representation of encapsulated RIPs in nanocapsules

As nanoparticles are able to cross the blood brain barrier, opening new perspectives to drug delivery into the brain, and since their nanosize allows access into the cell and in various cellular compartments, including the nucleus, the development of various RIP-based platforms has recently undergone a significant impetus (Fig. 3a and b).

For example, curcin [55] was successfully used in the construction of gold nanoparticles (AuNPs) conjugated with folate and anti-transferrin antibody, to achieve a dual targeted nano-formulation directed toward gliomas. AuNPs are nanoparticles (in the range of 10–15 nm) consisting of a gold core and a surface coating, modifiable to control particle stability, solubility and interaction with the biological environment. As well, the ability of the AuNP surface to bind to thiols and amines provides a convenient way to introduce reactive functional groups to conjugate therapeutic agents. In this case, AuNPs functionalized with PEG were preliminary conjugated to anti-transferrin antibodies and subsequently subjected to reaction with hydrazine-activated curcin. Authors revealed that this specific functionalized nanoconjugate minimized the nonspecific systemic spread of toxin molecules during circulation and maximize the efficiently of tumor-targeted drug delivery. Besides, once internalized, pH-sensitive hydrazone bonds guaranteed the maximum release of curcin from nanoconjugate with consequent efficacy of toxic action in the target glioma cancer colonies [55].

An additional example is represented by hybrid colloidal nanosystems, consisting of lipid polymeric components chemically amalgamated and highly compatible. Lipid-polymer hybrid nanoparticles are core-shell nanostructures comprising polymer cores and lipid/lipid-PEG shells, which exhibit characteristics of both polymeric nanoparticles and liposomes. In a recent work hybrid solid lipid nanoparticles were prepared by a modified lipid coacervation method. The latter is a procedure to forming a liquid rich in polymer phase in equilibrium with another liquid phase. In this case authors prepared hybrid solid lipid nanoparticles composed by 1,2-Distearoyl-sn-glycero-3-phosphoethanolamine-N-maleimide (PEG-2000) (DSPE-PEG-Mal), lecithin, stearic acid and folate. When these lipid based nanoparticles were conjugated to curcin, they became a nano-formulation selectively active against gliomas cells [56].

In a further work it has been described a different protein delivery strategy in which a new nanoconjugate has been obtained by combinatorial design of cationic lipid like materials, termed “*lipidoids*”, coupled with a reversible chemical protein engineering approach [57]. Preparation of lipidoids requires heating, in absence of solvent or catalysts, of commercially available amines with lipophilic acrylamides, acrylates, or epoxides. This simplified procedure allows building a structurally diverse library of lipidoids by varying the types of amines, and the lengths and types (acrylamide/acrylate/epoxide) of tails. Resulting crude products can be directly used for in vitro delivery of macromolecule of interest. Therefore, lipidoids offer two significant advantages: a simple and economical chemical synthesis and the possibility to develop libraries based on peculiar diversities (e.g. structure, physic-chemical properties, size etc.). In the case of RIPs, starting from a library of lipidoids synthesized through the ring opening reaction between 1,2-epoxyhexadecane and primary or secondary different aliphatic amines, a strong lipid-like nanoconjugated has been obtained using saporin-S6. This nanoconjugate was able to inhibit proliferation in vitro of various cancerous cell lines, with IC_50_ values greatly decreased compared to saporin alone, and was also found to abolish tumor growth in a mouse model of breast cancer [57].

Recently, it has been reported the emergent possibility to produce nanocapsules as efficient in vivo drug delivery system, Fig. 3c [58]. In this framework, also RIPs have proved to be optimal subject for this novel nanotechnology, as demonstrated in a recent paper where it has been reported the development of new drug formulations based on encapsulation of MAP 30 into chemically synthesized matrices of zirconium egg- (EPC) and soy-phosphatidylcholines (SPC). These matrices were obtained by mixing (EPC) or (SPC) lipids in ZrCl_4_ aqueous solution [59] and exhibited the plate like and granular aggregates with diameters of about 70 nm. Subsequent encapsulation of MAP30 produced strong toxic and antimicrobial nanoparticles and a release of RIP dependent on endogenous phospholipase A (PLA) activity [58].

A similar approach, by using type 1 RIP from *Mirabilis jalapa* L. leaves, has been recently used by Wicaksono et al. [60]. This RIP, easily degraded after administration, shows strong cytotoxicity on breast cancer cell lines and a negligible toxicity towards normal cells. In this work, the authors incorporated this type 1 RIP into nanoparticles conjugated with Anti-EpCAM antibodies and determined its selective cytotoxicity against T47D breast cancer cells. Nanoparticles were obtained by polyelectrolyte complexation using low viscosity chitosan (β-(1,4)-linked 2-amino-2-deoxy-β-D-glucose) and alginate (linear unbranched polymer of (1-4’)-linked β-D-mannuronic acid and α-L-guluronic acid residues) [61], then chemically conjugated with anti-EpCAM antibody by carbodiimide reaction.

All mentioned examples reveal that developing cancer targeted-therapy by using nanotechnology platform is very promising, especially with active-targeting nanoparticles [62]. It is widely reported that chemotherapeutic agents delivered systematically can be enter both tumor and critical organs (heart, lung, etc.) consequently determining patient’s suffering, noted as systemic toxicity. Systemic toxicity is the result of absorption and distribution of a chemoterapic agent to a site (organs, tissues, etc.) distant from its entry point of administration and it is the main cause of cancer drugs failure and of therapies who seek to differentiate between cancerous and normal cells [63].

Progressive development in active targeting smart nanoparticles and use of RIPs, in the construction of low dosage RIP based nanoconjugates, represent new possible future directions in the construction of innovative nanosystems aimed to mitigate or limiting system toxicity.

## Conclusion

Today the pharmacology is no longer exclusively oriented to the identification of “small molecules”, but also on searching for a second line of bio-based chemotherapeutic-conjugated, exploiting the engineering of recombinant proteins as well as the protein technology. In particular, for this reason, the pharmacology industries are exploring several options, such as specific cellular targets or new drug delivery methods.

**Fig. 4:**
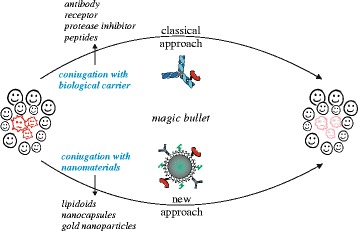
Schematic representation of classical and new approach to obtain RIP based conjugates. Classical approach is obtained with specific biological biomolecules, while a new approach is obtained by using novel nanomaterials

In both cases, RIPs proved to be good prototypes, due to their structures and toxicity. In this review, we tracked a path from first RIP based conjugates to recent RIP based nanoconstructs (Fig. 4), giving new emphasis to the study of these special and “ancient” molecules, which are essential natural defence elements in plant organisms, but at the same time excellent candidates for therapeutic applications in biomedical sciences.

## Abbreviations

ER, endoplasmic reticulum; PAP, pokeweed anti-viral protein; RIP(s), Ribosome-inactivating protein(s); rRNA, ribosomal ribonucleic acid

## References

[1] Endo Y, Tsurugi K (1987). RNA N-glycosidase activity of ricin A-chain. Mechanism of action of the toxic lectin ricin on eukaryotic ribosomes. J Biol Chem.

[2] Montanaro L, Sperti S, Mattioli A, Testoni G, Stirpe F (1975). Inhibition by ricin of protein synthesis in vitro. Inhibition of the binding of elongation factor 2 and of adenosine diphosphate-ribosylated elongation factor 2 to ribosomes. Biochem J.

[3] Santanche S, Bellelli A, Brunori M (1997). The unusual stability of saporin, a candidate for the synthesis of immunotoxins. Biochem Biophys Res Commun.

[4] Stirpe F, Battelli MG (2006). Ribosome-inactivating proteins: progress and problems. Cell Mol Life Sci.

[5] Nielsen K, Boston RS (2001). RIBOSOME-INACTIVATING PROTEINS: A Plant Perspective. Annu Rev Plant Physiol Plant Mol Biol.

[6] Jimenez P, Tejero J, Cordoba-Diaz D, Quinto EJ, Garrosa M, Gayoso MJ, Girbes T (2015). Ebulin from dwarf elder (*Sambucus ebulus* L.): a mini-review. Toxins (Basel).

[7] Girbes T, Citores L, Iglesias R, Ferreras JM, Munoz R, Rojo MA, Arias FJ, Garcia JR, Mendez E, Calonge M (1993). Ebulin 1, a nontoxic novel type 2 ribosome-inactivating protein from *Sambucus ebulus* L. leaves. J Biol Chem.

[8] Hey TD, Hartley M, Walsh TA (1995). Maize ribosome-inactivating protein (b-32). Homologs in related species, effects on maize ribosomes, and modulation of activity by pro-peptide deletions. Plant Physiol.

[9] Polito L, Bortolotti M, Mercatelli D, Battelli MG, Bolognesi A (2013). Saporin-S6: a useful tool in cancer therapy. Toxins (Basel).

[10] Park SW, Vepachedu R, Sharma N, Vivanco JM (2004). Ribosome-inactivating proteins in plant biology. Planta.

[11] Lord JM, Spooner RA (2011). Ricin trafficking in plant and mammalian cells. Toxins (Basel).

[12] Spooner RA, Lord JM (2015). Ricin Trafficking in Cells. Toxins (Basel).

[13] de Virgilio M, Lombardi A, Caliandro R, Fabbrini MS (2010). Ribosome-inactivating proteins: from plant defense to tumor attack. Toxins (Basel).

[14] Vago R, Marsden CJ, Lord JM, Ippoliti R, Flavell DJ, Flavell SU, Ceriotti A, Fabbrini MS (2005). Saporin and ricin A chain follow different intracellular routes to enter the cytosol of intoxicated cells. FEBS J.

[15] Polito L, Bortolotti M, Pedrazzi M, Mercatelli D, Battelli MG, Bolognesi A (2016). Apoptosis and necroptosis induced by stenodactylin in neuroblastoma cells can be completely prevented through caspase inhibition plus catalase or necrostatin-1. Phytomedicine.

[16] Barbieri L, Ciani M, Girbes T, Liu WY, Van Damme EJ, Peumans WJ, Stirpe F (2004). Enzymatic activity of toxic and non-toxic type 2 ribosome-inactivating proteins. FEBS Lett.

[17] Xiong SD, Yu K, Liu XH, Yin LH, Kirschenbaum A, Yao S, Narla G, DiFeo A, Wu JB, Yuan Y, Ho SM, Lam YW, Levine AC (2009). Ribosome-inactivating proteins isolated from dietary bitter melon induce apoptosis and inhibit histone deacetylase-1 selectively in premalignant and malignant prostate cancer cells. Int J Cancer.

[18] Wong JH, Ng TB, Cheung RC, Ye XJ, Wang HX, Lam SK, Lin P, Chan YS, Fang EF, Ngai PH, Xia LX, Ye XY, Jiang Y, Liu F (2010). Proteins with antifungal properties and other medicinal applications from plants and mushrooms. Appl Microbiol Biotechnol.

[19] Di Maro A, Pizzo E, Girbes T, Gopalakrishnakone P, Carlini RC, Ligabue-Braun R (2015). Biotechnological Potential of Ribosome Inactivating Proteins (RIPs). Plant Toxins.

[20] Gilabert-Oriol R, Weng A, Mallinckrodt B, Melzig MF, Fuchs H, Thakur M (2014). Immunotoxins constructed with ribosome-inactivating proteins and their enhancers: a lethal cocktail with tumor specific efficacy. Curr Pharm Des.

[21] Zheng Q, Xiong YL, Su ZJ, Zhang QH, Dai XY, Li LY, Xiao X, Huang YD (2013). Expression of curcin-transferrin receptor binding peptide fusion protein and its anti-tumor activity. Protein Expr Purif.

[22] Antignani A, Fitzgerald D (2013). Immunotoxins: the role of the toxin. Toxins (Basel).

[23] Becker N, Benhar I (2012). Antibody-Based Immunotoxins for the Treatment of Cancer. Antibodies.

[24] Spiess K, Jakobsen MH, Kledal TN, Rosenkilde MM. The future of antiviral immunotoxins. 2016. J Leukoc Biol. 2016;99(6):911-25. 10.1189/jlb.2MR1015-468R 26729815

[25] Li H, Gu C, Ren Y, Dai Y, Zhu X, Xu J, Li Y, Qiu Z, Zhu J, Zhu Y, Guan X, Feng Z. The efficacy of NP11-4-derived immunotoxin scFv-artesunate in reducing hepatic fibrosis induced by *Schistosoma japonicum* in mice. J Biomed Res. 2011;25(2):148–54. 10.1016/S1674-8301(11)60019-5 PMC3596707 23554683

[26] Hermanson GT (2008). Chapter 21 - Immunotoxin Conjugation Techniques. Bioconjugate Techniques (Second Edition).

[27] Hermanson GT (2008). Chapter 1 - Functional Targets. Bioconjugate Techniques (Second Edition).

[28] Dosio F, Brusa P, Cattel L (2011). Immunotoxins and anticancer drug conjugate assemblies: the role of the linkage between components. Toxins (Basel).

[29] Ghetie MA, May RD, Till M, Uhr JW, Ghetie V, Knowles PP, Relf M, Brown A, Wallace PM, Janossy G (1988). Evaluation of ricin A chain-containing immunotoxins directed against CD19 and CD22 antigens on normal and malignant human B-cells as potential reagents for in vivo therapy. Cancer Res.

[30] Youle RJ, Neville DM (1980). Anti-Thy 1.2 monoclonal antibody linked to ricin is a potent cell-type-specific toxin. Proc Natl Acad Sci U S A.

[31] Alewine C, Hassan R, Pastan I (2015). Advances in anticancer immunotoxin therapy. Oncologist.

[32] Maleki LA, Baradaran B, Majidi J, Mohammadian M, Shahneh FZ (2013). Future prospects of monoclonal antibodies as magic bullets in immunotherapy. Hum Antibodies.

[33] Tyagi N, Tyagi M, Pachauri M, Ghosh PC (2015). Potential therapeutic applications of plant toxin-ricin in cancer: challenges and advances. Tumour Biol.

[34] Tiller KE, Tessier PM (2015). Advances in Antibody Design. Annu Rev Biomed Eng.

[35] Farajnia S, Ahmadzadeh V, Tanomand A, Veisi K, Khosroshahi SA, Rahbarnia L (2014). Development trends for generation of single-chain antibody fragments. Immunopharmacol Immunotoxicol.

[36] Selbo PK, Weyergang A, Hogset A, Norum OJ, Berstad MB, Vikdal M, Berg K (2010). Photochemical internalization provides time- and space-controlled endolysosomal escape of therapeutic molecules. J Control Release.

[37] Cavallaro U, del Vecchio A, Lappi DA, Soria MR (1993). A conjugate between human urokinase and saporin, a type-1 ribosome-inactivating protein, is selectively cytotoxic to urokinase receptor-expressing cells. J Biol Chem.

[38] D’Cruz OJ, Waurzyniak B, Uckun FM (2004). Mucosal toxicity studies of a gel formulation of native pokeweed antiviral protein. Toxicol Pathol.

[39] Yang WH, Wieczorck M, Allen MC, Nett TM (2003). Cytotoxic activity of gonadotropin-releasing hormone (GnRH)-pokeweed antiviral protein conjugates in cell lines expressing GnRH receptors. Endocrinology.

[40] Lyu MA, Cao YJ, Mohamedali KA, Rosenblum MG (2012). Cell-targeting fusion constructs containing recombinant gelonin. Methods Enzymol.

[41] Deeks ED, Cook JP, Day PJ, Smith DC, Roberts LM, Lord JM (2002). The low lysine content of ricin A chain reduces the risk of proteolytic degradation after translocation from the endoplasmic reticulum to the cytosol. Biochemistry.

[42] Tamburino R, Pizzo E, Sarcinelli C, Poerio E, Tedeschi F, Ficca AG, Parente A, Di Maro A (2012). Enhanced cytotoxic activity of a bifunctional chimeric protein containing a type 1 ribosome-inactivating protein and a serine protease inhibitor. Biochimie.

[43] Di Maro A, Valbonesi P, Bolognesi A, Stirpe F, De Luca P, Siniscalco GG, Gaudio L, Delli Bovi P, Ferranti P, Malorni A, Parente A. Isolation and characterization of four type-1 ribosome-inactivating proteins, with polynucleotide:adenosine glycosidase activity, from leaves of *Phytolacca dioica* L. Planta. 1999;208(1):125–31. 10.1007/s004250050542 10213004

[44] Poerio E, Di Gennaro S, Di Maro A, Farisei F, Ferranti P, Parente A (2003). Primary structure and reactive site of a novel wheat proteinase inhibitor of subtilisin and chymotrypsin. Biol Chem.

[45] Sgambati V, Pizzo E, Mezzacapo MC, Di Giuseppe AM, Landi N, Poerio E, Di Maro A (2014). Cytotoxic activity of chimeric protein PD-L4UWSCI^(tr)^ does not appear be affected by specificity of inhibition mediated by anti-protease WSCI domain. Biochimie.

[46] Lv Q, Yang XZ, Fu LY, Lu YT, Lu YH, Zhao J, Wang FJ (2015). Recombinant expression and purification of a MAP30-cell penetrating peptide fusion protein with higher anti-tumor bioactivity. Protein Expr Purif.

[47] Lyons AM, Thiele TE (2010). Neuropeptide Y conjugated to saporin alters anxiety-like behavior when injected into the central nucleus of the amygdala or basomedial hypothalamus in BALB/cJ mice. Peptides.

[48] Minko T, Rodriguez-Rodriguez L, Pozharov V (2013). Nanotechnology approaches for personalized treatment of multidrug resistant cancers. Adv Drug Deliv Rev.

[49] Ahmad MZ, Alkahtani SA, Akhter S, Ahmad FJ, Ahmad J, Akhtar MS, Mohsin N, Abdel-Wahab BA (2016). Progress in nanotechnology-based drug carrier in designing of curcumin nanomedicines for cancer therapy: current state-of-the-art. J Drug Target.

[50] Uddin I, Venkatachalam S, Mukhopadhyay A, Usmani MA (2016). Nanomaterials in the pharmaceuticals: Occurrence, behaviour and applications.

[51] Daraee H, Eatemadi A, Abbasi E, Fekri AS, Kouhi M, Akbarzadeh A (2016). Application of gold nanoparticles in biomedical and drug delivery. Artif Cells Nanomed Biotechnol.

[52] Daraee H, Etemadi A, Kouhi M, Alimirzalu S, Akbarzadeh A (2016). Application of liposomes in medicine and drug delivery. Artif Cells Nanomed Biotechnol.

[53] Narayanaswamy R, Wang T, Torchilin VP (2016). Improving Peptide Applications Using Nanotechnology. Curr Top Med Chem.

[54] Avvakumova S, Colombo M, Tortora P, Prosperi D (2014). Biotechnological approaches toward nanoparticle biofunctionalization. Trends Biotechnol.

[55] Mohamed MS, Veeranarayanan S, Poulose AC, Nagaoka Y, Minegishi H, Yoshida Y, Maekawa T, Kumar DS (2014). Type 1 ribotoxin-curcin conjugated biogenic gold nanoparticles for a multimodal therapeutic approach towards brain cancer. Biochim Biophys Acta.

[56] Mohamed MS, Veeranarayanan S, Baliyan A, Poulose AC, Nagaoka Y, Minegishi H, Iwai S, Shimane Y, Yoshida Y, Maekawa T, Kumar DS (2014). Structurally distinct hybrid polymer/lipid nanoconstructs harboring a type-I ribotoxin as cellular imaging and glioblastoma-directed therapeutic vectors. Macromol Biosci.

[57] Wang M, Alberti K, Sun S, Arellano CL, Xu Q (2014). Combinatorially designed lipid-like nanoparticles for intracellular delivery of cytotoxic protein for cancer therapy. Angew Chem Int Ed Engl.

[58] Caizhen G, Yan G, Ronron C, Lirong Y, Panpan C, Xuemei H, Yuanbiao Q, Qingshan L. Zirconium phosphatidylcholine-based nanocapsules as an in vivo degradable drug delivery system of MAP30, a momordica anti-HIV protein. Int J Pharm. 2015;483(1–2):188–99. 10.1016/j.ijpharm.2015.02.021 25681721

[59] Qiao Y, Jian F, Bai Q (2008). Bioconjugation of zirconium uridine monophosphate: application to myoglobin direct electrochemistry. Biosens Bioelectron.

[60] Wicaksono PA, Name S, Martien R, Ismail H. Formulation and Cytotoxicity of Ribosome-Inactivating Protein *Mirabilis jalapa* L. Nanoparticles Using Alginate-Low Viscosity Chitosan Conjugated with Anti-Epcam Antibodies in the T47D Breast Cancer Cell Line. Asian Pac J Cancer Prev. 2016;17(4):2277–84. 10.7314/apjcp.2016.17.4.2277 27221930

[61] Hu CS, Chiang CH, Hong PD, Yeh MK (2012). Influence of charge on FITC-BSA-loaded chondroitin sulfate-chitosan nanoparticles upon cell uptake in human Caco-2 cell monolayers. Int J Nanomedicine.

[62] Lee WH, Loo CY, Young PM, Traini D, Mason RS, Rohanizadeh R (2014). Recent advances in curcumin nanoformulation for cancer therapy. Expert Opin Drug Deliv.

[63] Wang H, Yu J, Lu X, He X (2016). Nanoparticle systems reduce systemic toxicity in cancer treatment. Nanomedicine (London, England).

